# Association between the cardiometabolic index and non-alcoholic fatty liver disease: insights from a general population

**DOI:** 10.1186/s12876-022-02099-y

**Published:** 2022-01-12

**Authors:** Jiawang Zou, Hailan Xiong, Huimin Zhang, Chong Hu, Song Lu, Yang Zou

**Affiliations:** 1 Department of Cardiology, Jiujiang First People’s Hospital, Jiujiang, 332000 China; 2 Department of Cardiology, Jiangxi Provincial People’s Hospital, Nanchang, 330006 China; 3 Gastroenterology Department, Jiangxi Provincial People’s Hospital, Nanchang, 330006 China; 4 Jiangxi Cardiovascular Research Institute, Jiangxi Provincial People’s Hospital, Nanchang, 330006 China

**Keywords:** Cardiometabolic index, Non-alcoholic fatty liver disease, General population, CMI, NAFLD

## Abstract

**Background:**

Emerging evidence suggests that cardiometabolic index (CMI) is closely related to diabetes, hypertension, stroke, cardiovascular disease, and kidney disease, which implies that CMI has the value as an indicator of metabolic diseases. However, data on the relationships between CMI and non-alcoholic fatty liver disease (NAFLD) risks have not been reported. This study is designed to examine the association between CMI and NAFLD in the general population.

**Methods:**

The current study included 14,251 subjects whose CMI was the product of triglyceride/high-density lipoprotein cholesterol ratio and waist-to-height ratio. Linear regression was used to analyze the correlation between baseline information and CMI, logistic regression was used to study the relationship between CMI and NAFLD, and subgroup analysis was used to explore potential high-risk groups.

**Results:**

After adjusted for potential confounding factors, higher CMI was independently associated with NAFLD, in which every additional standard deviation (SD) of CMI increased the risk of NAFLD by 28% (OR 1.28 per SD increase, 95% CI 1.19–1.37, *P* for trend < 0.0001). There were also significant differences in CMI-related NAFLD risk among different ages and genders, in which the CMI-related NAFLD risk in young people was significantly higher than that in other age groups (OR = 2.63 per SD increase for young people, OR = 1.38 per SD increase for young and middle-aged people, OR = 1.18 per SD increase for middle-aged and elderly people; OR = 1.14 per SD increase for elderly people, *P* for interaction = 0.0010), and the CMI-related NAFLD risk in women was significantly higher than that in men (OR = 1.58 per SD increase for women, OR = 1.26 per SD increase for men, *P* for interaction = 0.0045).

**Conclusions:**

Current studies have found that after excluding potential confounding factors, higher CMI in the general population is independently associated with NAFLD risk.

**Supplementary Information:**

The online version contains supplementary material available at 10.1186/s12876-022-02099-y.

## Background

Non-alcoholic fatty liver disease (NAFLD) has escalated to a major public health problem worldwide [1, 2]. Similar to several other chronic diseases, NAFLD is extremely common in the general population. Recent epidemiological surveys indicate that about a quarter of adults worldwide suffer from NAFLD [3], and with the popularity of diabetes and obesity, diet and lifestyle changes, the aging of the population intensified, the prevalence of NAFLD is still constantly increasing. It is expected that in 10 years, the total number of NAFLD patients in the world will reach 1/3 of the total population, which will bring an inestimable disease burden to the whole mankind [3–6]. Furthermore, NAFLD is not benign static liver disease. Many studies have shown that NAFLD is a systemic, multi-system disease with continuous progression [7, 8]; It will affect the metabolic status of the body and act on the heart, brain, kidney, peripheral blood vessels and other target organs, resulting in adverse health consequences [8–10]. Therefore, it would be meaningful to identify the risk factors for NAFLD at an early stage.

The association of dyslipidemia, obesity and NAFLD has been well documented [11, 12]. Among them, the lipid ratio of triglyceride/high-density lipoprotein cholesterol (TG/HDL-C) is considered to be an excellent parameter for predicting the risk of NAFLD, which can better assess insulin resistance (IR) in the NAFLD population [13–15]. Waist-to-height ratio (WHtR) is a combination of waist circumference (WC) and height. Compared with the traditional simple body measurement index, it can better identify abdominal obesity, evaluate NAFLD, cardiac metabolic risk and a variety of non-communicable diseases [16–19]. More recently, Wakabayashi et al. developed a marker called cardiometabolic index (CMI) [20], calculated as WHtR multiplied by TG/HDL-C ratio, which combines lipid and obesity parameters into a simple and repeatable marker for the effective identification of diabetes [20, 21]. Additionally, several recent studies have found that CMI is closely related to hypertension, stroke, cardiovascular disease and kidney disease [22–25]. These results imply that CMI is significant as an indicator of metabolic diseases. However, research on the potential relationships between CMI and NAFLD risks has not been reported. This study is designed to examine the association between CMI and NAFLD in the general population.

## Methods

### Research design and data sources

NAGALA is a cohort study based on the general population, which has been going on since 1994. It aims to evaluate the risk factors of common chronic diseases in the general population, and provides useful research data for the early prevention and treatment of chronic diseases. The design scheme of NAGALA has been described in detail previously [26]. This study was a post hoc analysis of NAGALA, and a cross-sectional design was adopted according to the new research hypothesis, in which the available research data have been uploaded to the DRYAD database by Hamaguchi et al. [27]. This study extracted data from a general population of 20,944 people recruited in the NAGALA program who took a comprehensive health examination in Murakami Memorial Hospital from 2004 to 2015. According to the purpose of the study, subjects with alcoholic/viral hepatitis, diabetes, impaired fasting blood glucose, incomplete covariates and excessive drinking were excluded, and 14,251 subjects were included in the final analysis of this study. Since NAGALA's research scheme has been previously approved by the Murakami Memorial Hospital institutional review committee (IRB number: 2018-09-01), and the subject's identification information has been replaced with a check code, there is no needless for separate ethical approval and informed consent in this study.

### Clinical characteristics and biochemical parameters

Clinical characteristic parameters such as sex, height, weight, WC, age, smoking/drinking status, systolic/diastolic blood pressure (S/DBP) and habit of exercise were evaluated and recorded by trained medical workers with a standard questionnaire. The subjects were classified according to their weekly alcohol consumption and smoking status, and the drinking status was divided into three categories: non or less drinking (< 40 g/w), light drinking (40–139 g/w) and moderate drinking (140–209 g/w) [28]. Smoking status can be divided into three categories: non-smoking, past smoking and current smoking. Habit of exercise was divided into groups according to the subjects' weekly exercise, in which regular participation in any type of exercise at least once a week was considered to have exercise habits. Hematological samples used to analyze biochemical parameters were collected at least 8 h after fasting, and an automatic analyzer was used to analyze and determine gamma-glutamyl transferase (GGT), hemoglobin A1c (HbA1c), aspartate aminotransferase (AST), HDL-C, total cholesterol (TC), TG, fasting blood glucose (FPG) and alanine aminotransferase (ALT) according to the standard method.

### Diagnosis of NAFLD

As previously described [26], the diagnosis of NAFLD in the NAGALA project was carried out by ultrasound. Ultrasound examinations were performed by professional technicians, and ultrasound images were evaluated by experienced gastroenterologists without knowing the subjects' other examination and diagnostic information, based on the echo of the hepatorenal echo contrast, liver brightness, deep attenuation and vascular fuzzy four criteria for the score of 0 to 4 points, a comprehensive score greater than 2 points in the diagnosis of NAFLD [29].

### Statistical analysis

The statistical software Empower Stats (version 2.0) and R language (version 3.4.3) were used for data analysis. To further understand the correlation between CMI and NAFLD, this study took CMI as a continuous variable and a categorical variable for correlation analysis. The baseline characteristics of all patients were stratified by CMI quartile, and categorical variables and continuous variables were described as percentages or mean (standard deviation: SD) or median (interquartile range) according to the type and distribution pattern of variables. Chi-square test or one-way ANOVA or rank-sum test were used for inter-group comparison, and linear regression was used to check the association between baseline variables and CMI. Univariate and multivariate logistic regression models were then used to determine whether CMI was independently associated with NAFLD, and the results show that the odds ratio (OR) and the corresponding 95% confidence interval (CI). In the multivariate regression analysis, we ran a total of three models. According to the STROBE statement [30], we adopted different variable adjustment strategies in the three models. Model one, as a fine-tuning model, only adjusted the non-collinear general demographic variables (Additional file 1: Table S1), including sex, age, body mass index (BMI), drinking status and smoking status. In model two, the non-collinear covariates (that affecting the matching risk of CMI and NAFLD by more than 10% were regarded as confounding variables) were adjusted [31]. Model three further adjusted the non-collinear covariates that were statistically related to NAFLD in univariate analysis based on Model two. Additionally, we also conducted an exploratory stratified analysis among people of different ages, genders, BMI and whether or not to preserve exercise habits, and checked whether the effect of CMI on NAFLD was different among different subgroups by the likelihood ratio test. The receiver operating characteristic (ROC) curve was constructed to evaluate the accuracy of WC, height, TG, HDL-C and CMI in determining NAFLD, and the best cut-off value of CMI was calculated. In addition, we further used restricted cubic splines to flexibly model the association between the continuous variable CMI and NAFLD.

## Results

### Subject characteristics

Overall 14,251 subjects were enrolled in this study, and the study population was quartered according to the CMI quartile. Table 1 summarizes the clinical and biochemical characteristics of the study population. The prevalence rates of NAFLD in the four groups were 1.26%, 5.96%, 17.56% and 45.52%, respectively. There were significant differences in parameters and indicators among the CMI groups (all *P* < 0.0001). Compared with the group with low CMI, the group with high CMI was older, had more people with smoking and drinking status, and had higher anthropometric parameters such as height, weight, BMI, WC and blood pressure, while the serological parameters except HDL-C increased gradually with the increase of CMI.

**Table 1:** Characteristics of the subject

	CMI quartile				*P*-value
	Q1 (0.01–0.13)	Q2 (0.13–0.23)	Q3 (0.23–0.43)	Q4 (0.43–7.99)	
No. of subjects	3561	3560	3560	3561	
Age, years	40.00 (35.00, 46.00)	42.00 (36.00, 49.00)	44.00 (38.00, 51.00)	45.00 (39.00, 52.00)	< 0.001
Sex					< 0.001
Women	2792 (78.40%)	2094 (58.82%)	1320 (37.08%)	634 (17.80%)	
Men	769 (21.60%)	1466 (41.18%)	2240 (62.92%)	2927 (82.20%)	
Weight, kg	51.30 (46.80, 56.70)	55.90 (50.00, 62.90)	61.70 (55.00, 68.30)	69.00 (62.10, 76.30)	< 0.001
Height, cm	161.78 ± 7.49	163.61 ± 8.59	165.66 ± 8.52	168.12 ± 7.94	< 0.001
BMI, kg/m^2^	19.95 ± 2.12	21.18 ± 2.48	22.54 ± 2.69	24.59 ± 3.10	< 0.001
WC, cm	69.35 ± 6.37	73.44 ± 7.34	77.87 ± 7.50	84.06 ± 7.84	< 0.001
NAFLD	45 (1.26%)	212 (5.96%)	625 (17.56%)	1621 (45.52%)	< 0.001
WHtR	0.43 ± 0.04	0.45 ± 0.04	0.47 ± 0.04	0.50 ± 0.05	< 0.001
ALT, IU/L	14.00 (11.00, 17.00)	15.00 (12.00, 19.00)	17.00 (13.00, 23.00)	23.00 (17.00, 32.00)	< 0.001
AST, IU/L	16.00 (13.00, 19.00)	16.00 (13.00, 20.00)	17.00 (14.00, 21.00)	19.00 (16.00, 24.00)	< 0.001
GGT, IU/L	12.00 (10.00, 15.00)	13.00 (10.00, 17.00)	16.00 (12.00, 22.00)	21.00 (15.00, 31.00)	< 0.001
HDL-C, mmol/L	1.84 ± 0.37	1.57 ± 0.29	1.35 ± 0.25	1.08 ± 0.21	< 0.001
TC, mmol/L	4.85 ± 0.81	5.00 ± 0.80	5.18 ± 0.86	5.47 ± 0.87	< 0.001
TG, mmol/L	0.38 (0.30, 0.45)	0.60 (0.52, 0.70)	0.87 (0.75, 1.00)	1.48 (1.21, 1.91)	< 0.001
TG/HDL-C ratio	0.22 (0.17, 0.26)	0.39 (0.34, 0.44)	0.65 (0.56, 0.76)	1.33 (1.06, 1.84)	< 0.001
FPG, mmol/L	4.95 ± 0.39	5.08 ± 0.40	5.21 ± 0.38	5.34 ± 0.37	< 0.001
HbA1c, %	5.14 ± 0.30	5.15 ± 0.31	5.19 ± 0.33	5.24 ± 0.34	< 0.001
SBP, mmHg	107.26 ± 12.71	111.51 ± 13.66	115.81 ± 14.41	121.15 ± 14.73	< 0.001
DBP, mmHg	66.22 ± 8.97	69.25 ± 9.51	72.54 ± 9.95	76.47 ± 10.16	< 0.001
Habit of exercise					< 0.001
No	2886 (81.04%)	2941 (82.61%)	2934 (82.42%)	3013 (84.61%)	
Yes	675 (18.96%)	619 (17.39%)	626 (17.58%)	548 (15.39%)	
Drinking status					< 0.001
Nor or less	3185 (89.44%)	2983 (83.79%)	2866 (80.51%)	2768 (77.73%)	
Light	298 (8.37%)	438 (12.30%)	505 (14.19%)	513 (14.41%)	
Moderate	78 (2.19%)	139 (3.90%)	189 (5.31%)	280 (7.86%)	
Smoking status					< 0.001
Nor	2881 (80.90%)	2437 (68.46%)	1929 (54.19%)	1495 (41.98%)	
Past	388 (10.90%)	567 (15.93%)	745 (20.93%)	856 (24.04%)	
Current	292 (8.20%)	556 (15.62%)	886 (24.89%)	1210 (33.98%)	

Values were expressed as mean (SD) or medians (quartile interval) or n (%)

NAFLD, non-alcoholic fatty liver disease; BMI, body mass index; WC, Waist circumference; WHtR, waist-to-height ratio; ALT, alanine aminotransferase; AST, aspartate aminotransferase; GGT, gamma-glutamyl transferase; HDL-C, high-density lipoprotein cholesterol; TC, total cholesterol; TG, triglyceride; HbA1c, hemoglobin A1c; FPG, fasting plasma glucose; SBP, systolic blood pressure; DBP, Diastolic blood pressure; CMI, cardiometabolic index

### Correlation analysis between CMI and baseline variables

The correlation between CMI and baseline variables was evaluated using linear regression, and the results are summarized in Table 2. It is obvious from the table that all the baseline variables have a linear correlation with CMI, in which habit of exercise, HDL-C has a negative correlation with CMI, while other variables have a positive correlation with CMI. It is worth noting that the related variables of blood glucose and blood lipid metabolism were strongly correlated with CMI, and the correlation between WHtR and CMI is the strongest. This finding hints at a possibility that CMI has the value as an index of metabolic diseases; Additionally, these variables significantly correlated with CMI may be cofactors for the association between CMI and NAFLD.

**Table 2:** Association between CMI and baseline variables

	Statistics	Effect size (β)	*P*-value
Sex			
Women	6840 (48.00%)	Ref	
Men	7411 (52.00%)	0.26 (0.25, 0.28)	< 0.0001
Age	43.53 ± 8.89	0.01 (0.00, 0.01)	< 0.0001
Weight	60.26 ± 11.61	0.01 (0.01, 0.02)	< 0.0001
Height	164.80 ± 8.48	0.01 (0.01, 0.01)	< 0.0001
BMI	22.06 ± 3.14	0.06 (0.05, 0.06)	< 0.0001
WC	76.19 ± 9.10	0.02 (0.02, 0.02)	< 0.0001
WHtR	0.46 ± 0.05	3.27 (3.16, 3.38)	< 0.0001
Habit of exercise			
No	11,781 (82.67%)	Ref	
Yes	2470 (17.33%)	−0.03 (−0.04, −0.01)	0.0008
ALT	19.76 ± 14.47	0.01 (0.01, 0.01)	< 0.0001
AST	18.23 ± 8.67	0.01 (0.01, 0.01)	< 0.0001
GGT	19.13 ± 16.13	0.01 (0.01, 0.01)	< 0.0001
HDL-C	1.46 ± 0.40	−0.54 (−0.55, −0.52)	< 0.0001
TC	5.12 ± 0.87	0.09 (0.09, 0.10)	< 0.0001
TG	0.89 ± 0.63	0.56 (0.56, 0.56)	< 0.0001
TG/HDL-C ratio	0.73 ± 0.74	0.51 (0.51, 0.51)	< 0.0001
FPG	5.15 ± 0.41	0.26 (0.25, 0.28)	< 0.0001
HbA1c	5.18 ± 0.32	0.12 (0.10, 0.14)	< 0.0001
Drinking status			
Nor or less	11,805 (82.84%)	Ref	
Light	1758 (12.34%)	0.06 (0.04, 0.08)	< 0.0001
Moderate	688 (4.83%)	0.11 (0.08, 0.14)	< 0.0001
Smoking status			
Nor	8746 (61.37%)	Ref	
Past	2559 (17.96%)	0.14 (0.12, 0.15)	< 0.0001
Current	2946 (20.67%)	0.22 (0.21, 0.24)	< 0.0001
SBP	113.93 ± 14.82	0.01 (0.01, 0.01)	< 0.0001
DBP	71.12 ± 10.38	0.01 (0.01, 0.01)	< 0.0001

OR, odds ratios; other abbreviations as in Table 1

### Association between CMI and NAFLD

Table 3 shows the results of univariate logical regression analysis. In univariate analysis, except for drinking status, all the covariables were significantly correlated with NAFLD, and the correlation between CMI and NAFLD was the highest (OR 19.64, 95% CI 16.97–22.73); These covariates related to NAFLD will be incorporated into the multivariable model as confounding factors. In order to be consistent with the results of previous studies [22–25], CMI was also converted into Z scores for analysis in this study. In the multivariate regression analysis (Table 4), after adjusting the general demographic variables, model one found that CMI was positively correlated with NAFLD (OR 1.67 per SD increase, 95% CI 1.58–1.77), and the NAFLD risk gradually increased among the CMI quartiles [OR (Q1:1, Q2:4.95, Q3:16.64, Q4:65.29, *P* for trend < 0.0001). Model two adjusted for non-collinear covariates that altered the matching risk of CMI and NAFLD by more than 10%, the positive correlation weakened slightly (OR 1.28 per SD increase, 95% CI 1.20–1.37, *P* for trend < 0.0001). After further adjusting the non-collinear covariates related to NAFLD in univariate analysis (Model three: OR 1.28 per SD increase, 95% CI 1.19–1.37, *P* for trend < 0.0001), the positive correlation between them remained stable. In short, CMI is an independent risk factor for NAFLD.

**Table 3:** Univariate analysis of the association between NAFLD and baseline variables

	OR (95%CI)	*P*-value
Sex		
Women	Ref	
Men	5.02 (4.51, 5.58)	< 0.0001
Age	1.02 (1.01, 1.02)	< 0.0001
Weight	1.13 (1.12, 1.14)	< 0.0001
Height	1.06 (1.05, 1.06)	< 0.0001
BMI	1.65 (1.61, 1.68)	< 0.0001
WC	1.20 (1.19, 1.21)	< 0.0001
WHtR (Per SD)	4.06 (3.82., 4.31)	< 0.0001
Habit of exercise		
No	Ref	
Yes	0.82 (0.72, 0.92)	0.0008
ALT	1.10 (1.10, 1.11)	< 0.0001
AST	1.09 (1.08, 1.10)	< 0.0001
GGT	1.04 (1.04, 1.04)	< 0.0001
HDL-C	0.05 (0.05, 0.06)	< 0.0001
TC	1.65 (1.57, 1.73)	< 0.0001
TG	4.65 (4.30, 5.03)	< 0.0001
TG/HDL-C	3.92 (3.65, 4.21)	< 0.0001
FPG	6.91 (6.13, 7.78)	< 0.0001
HbA1c	4.42 (3.84, 5.08)	< 0.0001
Drinking status		
Nor or less	Ref	
Light	0.90 (0.79, 1.04)	0.1442
Moderate	1.12 (0.92, 1.36)	0.2731
Smoking status		
Nor	Ref	
Past	2.12 (1.91, 2.37)	< 0.0001
Current	1.93 (1.73, 2.14)	< 0.0001
SBP	1.05 (1.05, 1.06)	< 0.0001
DBP	1.08 (1.07, 1.08)	< 0.0001
CMI	19.64 (16.97, 22.73)	< 0.0001
CMI Z score	3.08 (2.91, 3.25)	< 0.0001

OR, odds ratios; other abbreviations as in Table 1

**Table 4:** Association between CMI and NAFLD in different models

	OR (95%CI)					*P* for trend
	Multivariable Analysis	Q1	Q2	Q3	Q4	
Model one	1.67 (1.58, 1.77)	Ref	2.60 (1.86, 3.63)	5.09 (3.70, 7.00)	11.05 (8.03, 15.22)	< 0.0001
Model two	1.28 (1.20, 1.37)	Ref	2.03 (1.44, 2.86)	3.24 (2.29, 4.57)	5.53 (3.80, 8.04)	< 0.0001
Model three	1.28 (1.19, 1.37)	Ref	2.11 (1.49, 3.01)	3.48 (2.44, 4.96)	5.55 (3.77, 8.19)	< 0.0001

OR: odds ratios; CMI: cardiometabolic index; NAFLD: non-alcoholic fatty liver disease

Model one adjusted for: sex, age, BMI, drinking status and smoking status

Model two adjusted for: sex, BMI, WHtR, GGT, HDL-C, TC, FPG, drinking status and SBP

Model three adjusted for: sex, age, BMI, ALT, AST, height, WHtR, GGT, habit of exercise, HDL-C, TC, FPG, drinking status, smoking status, SBP and HbA1c

### Subgroup analysis

In the subgroup analysis, the researchers further evaluated whether CMI and NAFLD differed between different populations by stratification analysis and interactivity tests. The age and BMI were stratified according to the commonly used clinical cutoff points, and the results of stratified analysis and interactive test suggested that (Table 5): there were significant differences between CMI and NAFLD in different ages and genders (*P* for interaction < 0.05), but no significant statistical differences were found in BMI and habit of exercise stratification (*P* for interaction > 0.05). Of note, this study found that the CMI-related NAFLD risk of young people was significantly higher than that of other age groups (OR = 2.63 per SD increase for young people, OR = 1.38 per SD increase for young and middle-aged people, OR = 1.18 per SD increase for middle-aged and elderly people; OR = 1.14 per SD increase for elderly people, *P* for interaction = 0.0010), and the CMI-related NAFLD risk of women was significantly higher than that of men (OR = 1.58 per SD increase for women, OR = 1.26 per SD increase for men, *P* for interaction = 0.0045).

**Table 5:** Stratified associations between CMI and NAFLD by age, sex, BMI, and habit of exercise

Subgroup	No. of participants	Unadjusted OR (95% CI)	Adjusted OR (95% CI)	*P* for interaction
Age (years)				0.0010
18–30	645	9.72 (5.49, 17.21)	2.63 (1.45, 4.78)	
31–45	8204	3.75 (3.47, 4.07)	1.38 (1.27, 1.51)	
46–60	4906	2.42 (2.23, 2.62)	1.18 (1.09, 1.29)	
> 60	487	1.66 (1.35, 2.04)	1.14 (0.93, 1.40)	
Sex				0.0045
Women	6840	5.23 (4.50, 6.07)	1.58 (1.34, 1.87)	
Men	7402	2.26 (2.13, 2.39)	1.26 (1.17, 1.35)	
BMI (kg/m^2^)				0.1705
< 18.5	1543	4.51 (0.80, 25.27)	1.83 (0.21, 16.01)	
≥ 18.5, < 24	9334	2.37 (2.19, 2.57)	1.33 (1.22, 1.46)	
≥ 24, < 28	2721	1.75 (1.61, 1.89)	1.22 (1.12, 1.32)	
≥ 28	644	2.26 (1.78, 2.87)	1.50 (1.19, 1.89)	
Habit of exercise				0.0851
Yes	2468	3.19 (3.00, 3.39)	1.31 (1.22, 1.41)	
No	11,774	2.54 (2.22, 2.89)	1.18 (1.05, 1.32)	

OR: odds ratios; other abbreviations as in Table 1

Adjusted for: sex, BMI, WHtR, GGT, HDL-C, TC, FPG, drinking status and SBP

### Determine the best cutoff value for NAFLD

**Fig. 1:**
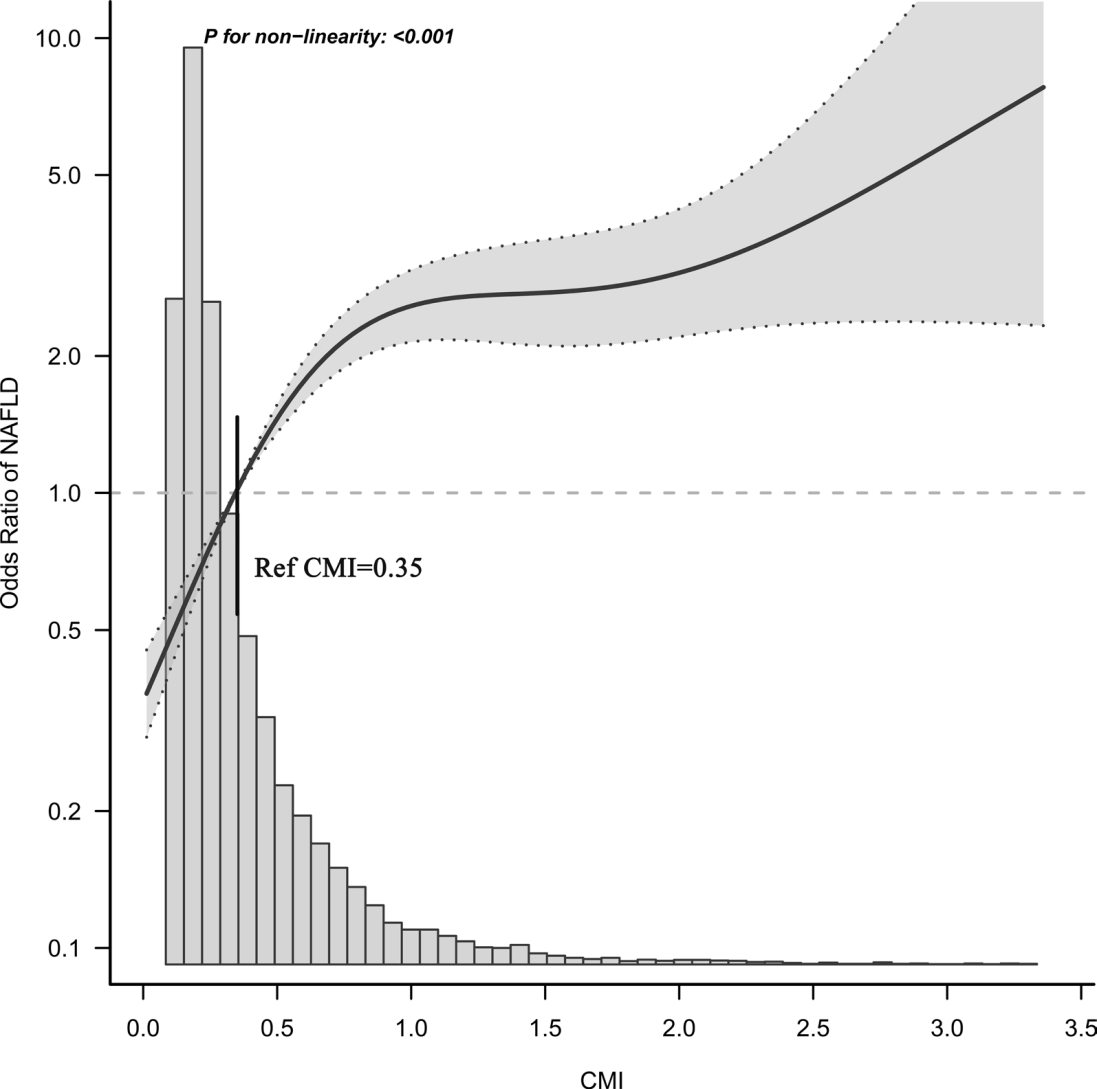
Odds ratios of NAFLD based on CMI. Model was adjusted for sex, age, BMI, ALT, AST, height, WHtR, GGT, habit of exercise, HDL-C, TC, FPG, drinking status, smoking status, SBP and HbA1c. Data were fitted by a restricted cubic spline logistic regression model. The 95% confidence intervals are indicated by the dashed line

ROC analysis was performed to calculate WC, height, TG, HDL-C and CMI for determining the area under the curve of NAFLD. The results showed that among these parameters, the accuracy of WC in determining NAFLD was the highest, followed by CMI, and the best cutoff value of CMI in determining NAFLD was 0.3241 (Table 6). In order to further verify whether the cutoff value used by CMI to determine NAFLD was stable, we continue to use restricted cubic splines to simulate the association between CMI and NAFLD. As shown in Fig. 1, when CMI was about 0.35, it may be a potential threshold point for NAFLD risk.

**Table 6:** The AUC for each index to discriminate NAFLD

	AUC	95% CI low	95% CI upp	Cutoff value	Specificity	Sensitivity
CMI	0.8359	0.8279	0.8439	0.3241	0.7409	0.7787
WC	0.8610	0.8539	0.8681	79.6500	0.7571	0.8085
Height	0.6395	0.6280	0.6510	164.1500	0.5244	0.7164
TG	0.7969	0.7877	0.8061	0.8411	0.6799	0.7698
HDL-C	0.7587	0.7489	0.7685	1.3408	0.6481	0.7543

AUC, area under the curve; CI, confidence interval; HDL-C, high-density lipoprotein cholesterol; LDL-C, low-density lipoprotein cholesterol; NAFLD, non-alcoholic fatty liver disease; WC, Waist circumference; TG, triglyceride; CMI, cardiometabolic index

## Discussion

In this cross-sectional study based on the general population, we examined the association between CMI and NAFLD, and found a strong positive correlation between higher CMI and the risk of NAFLD, independent of metabolic factors such as blood glucose and lipids. As far as we know, this study provides the first evidence of the relationship between CMI and NAFLD. The findings of this study provide new ideas for the risk assessment of NAFLD and are of great significance.

NAFLD is characterized by steatosis of the liver, which promotes the occurrence and progression of NAFLD with the participation of inflammation, oxidative stress and IR [11, 32]. In many previous observational studies, various lipid abnormalities have received widespread attention because of their significant increase in the risk of NAFLD [11, 33], among which the disorder of lipoproteins rich in TG is considered to be a key factor in cardiovascular risk in patients with NAFLD [34]. In this context, various lipid parameters and lipid ratios have been widely explored and studied, among which TG/HDL-C ratio seems to be a better predictor of NAFLD [13, 14], which may be related to IR. Several recent studies have shown that TG/HDL-C ratio is significantly associated with IR in whites and yellow race, and is considered to be an effective clinical alternative marker for IR [15, 35, 36], while it seems inappropriate to use TG/HDL-C ratio to predict IR in Hispanics and African Americans [15]. At present, drugs for the treatment of NAFLD with dyslipidemia as the entry point are also being extensively studied [11, 37, 38].

Obesity is the main risk factor for the prompt increase of NAFLD in recent years, and most obesity-related parameters are also important risk factors for NAFLD [12, 39]. Both BMI and WC are the most commonly used anthropometric indicators to evaluate obesity. Their measurements are simple, convenient and reproducible, so they have been widely used in the clinic. However, BMI has an obvious limitation, it may not be able to distinguish excess adipose tissue [40, 41], while WC is an excellent indicator of visceral obesity. In recent years, more and more studies have found that WHtR, which combines height and WC, is a better alternative indicator of obesity, and it is also a better determinant of NAFLD risk than BMI and WC [16, 19].

CMI is a newly developed clinical indicator that is a marker of the combination of WHtR and TG/HDL-C ratio. It was first reported by Wakabayashi et al. in 2015 that it has a significant advantage in the evaluation of diabetes [20]. Subsequent studies have further found that CMI is closely associated with hypertension, stroke, cardiovascular disease, and kidney disease [22–25], suggesting that CMI has value as an indicator of metabolic diseases. However, there is currently no data on the association between CMI and NAFLD. In this sense, this study verified the correlation between CMI and NAFLD in the general population for the first time based on a large sample size.

In the subgroup analysis, the study also assessed whether there were differences in the association between CMI and NAFLD in different populations. The result showed that there were significant differences between CMI and NAFLD in different ages and genders, but no significant statistical differences were found in BMI and habit of exercise stratification. Notably, women were found to have a higher risk of CMI-related NAFLD than men in this study, while similar results appear to have been found in several other CMI-related studies [20, 21, 23, 24]. In an earlier study by Wakabayashi et al., they evaluated 10,196 subjects who took a health check and found that the association between CMI and diabetes was stronger in women than in men (OR: 14.61 for women, 5.38 for men) [20]. Sun and his team also found this gender difference in the Chinese population in a follow-up study [21]. And in 2017, Wang and his colleagues found that CMI also differed between the sexes in cerebrovascular disease and hypertension, with CMI significantly more strongly associated with stroke and hypertension in women than in men [23, 24]. Taken together, CMI seems to be a more suitable marker for metabolic-related diseases in women. In addition, this study also found that among young people, the risk of CMI-related NAFLD was significantly higher than that of other age groups. In order to explain this special phenomenon, we further describe the baseline variables according to age (Additional file 1: Table S2), and it is worth noting that in this study, young NAFLD patients have higher BMI, WC, TG/HDL-C ratio and CMI values, while relatively few people maintain exercise habits. With the current obesity epidemic, young people in today's society may already be overweight or obese in childhood [3, 42]. Furthermore, with the accelerated development of social information, the aggravation of social aging, and accompanied by a wide range of unhealthy living habits among young people, young people also begin to suffer from NAFLD and other metabolic diseases prematurely [43–45]. The results of this study suggest that young people should enhance physical exercise to avoid obesity.

## Research strengths and limitations

The advantage of this study will be reflected in that it conducted an epidemiological survey of the general population in the case of large sample size, and subgroup analysis was used to check whether there were differences between CMI and NAFLD in different populations. As far as we know, this study reports the data of the relationship between CMI and NAFLD for the first time.

This study also has some limitations. First, the diagnosis of NAFLD in this study was evaluated by abdominal ultrasound; it is undeniable that abdominal ultrasound may not be sensitive enough for mild fatty liver [46]. However, for large-scale epidemiological investigations, liver biopsy does not seem to be a suitable choice, which is an urgent problem to be solved at present. Second, the current study adopts a cross-sectional design, so it is impossible to explain whether there is a causality relationship between CMI and NAFLD. Third, due to the lack of some parameters for calculating the score of liver fibrosis in this study, it is impossible to analyze the association between CMI and liver fibrosis in the current study. Fourth, because subjects with diabetes and impaired fasting blood glucose were excluded from the study, there may be a certain selection bias. Finally, although the current research has adopted strict standards to identify confounding factors, there are still some potential residual confounding variables because they are unmeasurable or not collected in this data set.

## Conclusion

Current studies have shown that there is a strong positive correlation between higher CMI and the risk of NAFLD in the general population, independent of traditional risk factors. Additionally, the results of subgroup analysis showed that the CMI-related NAFLD risk of young people was significantly higher than that of other age groups, and the CMI-related NAFLD risk of women was significantly higher than that of men. The content of this study suggests that regular examination and evaluation of CMI may help control the occurrence of NAFLD, and young people should strengthen physical exercise and pay attention to body obesity.

## Supplementary Information

**Additional file 1.** **Supplementary Table 1:** Collinearity diagnostics steps.

## Data Availability

The datasets analyzed during the current study are available in the [DRYAD] repository (https://datadryad.org/stash/dataset/doi:10.5061%2Fdryad.8q0p192).

## Abbreviations

CMI: Cardiometabolic index
NAFLD: Non-alcoholic fatty liver disease
SD: Standard deviation
TG/HDL-C: Triglyceride/high-density lipoprotein cholesterol
IR: Insulin resistance
WHtR: Waist-to-height ratio
WC: Waist circumference
ALT: Alanine aminotransferase
AST: Aspartate aminotransferase
GGT: Gamma-glutamyl transferase
TC: Total cholesterol
HbA1c: Hemoglobin A1c
FPG: Fasting blood glucose
OR: Odds ratio
CI: Confidence interval
BMI: Body mass index
S/DBP: Systolic/diastolic blood pressure
