# Supplementary material for: Association between the cardiometabolic index and non-alcoholic fatty liver disease: insights from a general population
Source: BMC Gastroenterol. 2022 Jan 12;22:20. doi: 10.1186/s12876-022-02099-y (PMC8756663; doi:10.1186/s12876-022-02099-y)
Supplement: Supplementary file 1 — Additional file 1. Supplementary Table 1: Collinearity diagnostics steps. [file 12876_2022_2099_MOESM1_ESM.docx]

Additional file 1: Table S1. Collinearity diagnostics steps.

|  | Step 1 | Step 2 | Step 3 | Step 4 | Step 5 | Step 6 |
| --- | --- | --- | --- | --- | --- | --- |
| CMI | 159.3 | 157.6 | 12.1 | 12.1 | 1.9 | 1.9 |
| Sex | 3.3 | 3.3 | 3.3 | 3.2 | 3.2 | 3.2 |
| Age | 1.4 | 1.4 | 1.4 | 1.4 | 1.4 | 1.3 |
| BMI | 229.1 | 99.8 | 99.6 | 4.9 | 4.9 | 4.9 |
| WC | 1214.4 | NA | NA | NA | NA | NA |
| Weight | 426.7 | 170.9 | 170.1 | NA | NA | NA |
| Height | 92.6 | 52.1 | 51.9 | 2.5 | 2.5 | 2.5 |
| WHtR | 960 | 5.8 | 4.8 | 4.7 | 4.7 | 4.7 |
| ALT | 4.1 | 4.1 | 4.1 | 4.1 | 4.1 | 4.1 |
| AST | 3.3 | 3.3 | 3.3 | 3.3 | 3.3 | 3.3 |
| GGT | 1.5 | 1.5 | 1.5 | 1.5 | 1.4 | 1.4 |
| Habit of exercise | 1 | 1 | 1 | 1 | 1 | 1 |
| HDL-C | 2.3 | 2.3 | 2 | 2 | 2 | 2 |
| TC | 1.5 | 1.5 | 1.5 | 1.5 | 1.4 | 1.4 |
| TG | 13.6 | 13.6 | 11.2 | 11.2 | NA | NA |
| TG/HDL-C ratio | 181.1 | 179.6 | NA | NA | NA | NA |
| FPG | 1.4 | 1.4 | 1.4 | 1.3 | 1.3 | 1.3 |
| Drinking status | 1.2 | 1.2 | 1.2 | 1.2 | 1.2 | 1.2 |
| Smoking status | 1.4 | 1.4 | 1.4 | 1.4 | 1.4 | 1.4 |
| SBP | 5.5 | 5.5 | 5.5 | 5.5 | 5.5 | 1.4 |
| DBP | 5.6 | 5.6 | 5.6 | 5.6 | 5.6 | NA |

VIF = 1/(1-R^2^). Abbreviations as in Table ​1.

Additional file 1: Table S2. Baseline characteristics of patients with NAFLD grouped by age.

|  | Age group | | | |  |
| --- | --- | --- | --- | --- | --- |
|  | >60 years | 46-60 years | 31-45 years | 18-30 years | *P*-value |
| No. of subjects | 81 | 993 | 1392 | 41 |  |
| Sex |  |  |  |  | <0.001 |
| Women | 18 (22.22%) | 281 (28.30%) | 172 (12.36%) | 7 (17.07%) |  |
| Men | 63 (77.78%) | 712 (71.70%) | 1220 (87.64%) | 34 (82.93%) |  |
| BM, kg/m^2^ | 24.47 (2.10) | 24.98 (2.82) | 25.91 (3.25) | 26.34 (4.93) | <0.001 |
| WC, cm | 84.91 (6.71) | 85.15 (7.41) | 86.62 (7.96) | 86.42 (10.61) | <0.001 |
| Weight, kg | 65.30 (61.50-71.10) | 68.50 (61.60-74.90) | 73.70 (67.30-81.73) | 74.00 (66.70-84.30) | <0.001 |
| Height, cm | 163.35 (8.02) | 165.56 (8.23) | 170.00 (7.00) | 170.17 (7.60) | <0.001 |
| WHtR | 0.52 (0.04) | 0.51 (0.04) | 0.51 (0.05) | 0.51 (0.06) | 0.020 |
| ALT, U/L | 24.00 (18.00-29.00) | 24.00 (19.00-32.00) | 30.00 (22.00-44.00) | 33.00 (20.00-44.00) | <0.001 |
| AST, U/L | 22.00 (17.00-25.00) | 20.00 (16.00-24.00) | 21.00 (17.00-27.00) | 21.00 (16.00-26.00) | <0.001 |
| GGT, U/L | 22.00 (16.00-29.00) | 21.00 (15.00-30.00) | 24.00 (18.00-35.00) | 22.00 (15.00-28.00) | <0.001 |
| HDL-C, mmol/L | 1.25 (0.34) | 1.21 (0.31) | 1.16 (0.26) | 1.10 (0.26) | <0.001 |
| TC, mmol/L | 5.59 (0.75) | 5.54 (0.86) | 5.37 (0.87) | 5.16 (0.86) | <0.001 |
| TG, mmol/L | 1.13 (0.76-1.59) | 1.23 (0.87-1.73) | 1.25 (0.88-1.83) | 1.12 (0.82-1.51) | 0.237 |
| TG/HDL-C ratio | 0.98 (0.55-1.54) | 1.05 (0.66-1.62) | 1.12 (0.71-1.77) | 0.98 (0.74-1.48) | 0.048 |
| CMI | 0.48 (0.28-0.81) | 0.54 (0.34-0.83) | 0.57 (0.35-0.92) | 0.55 (0.36-0.77) | 0.385 |
| FPG, mmol/L | 5.45 (0.35) | 5.41 (0.37) | 5.38 (0.36) | 5.35 (0.31) | 0.053 |
| HbA1c, % | 5.38 (0.33) | 5.34 (0.34) | 5.27 (0.32) | 5.26 (0.42) | <0.001 |
| SBP, mmHg | 125.20 (15.17) | 123.11 (15.86) | 123.46 (14.09) | 125.43 (12.72) | 0.502 |
| DBP, mmHg | 78.38 (9.37) | 78.25 (10.58) | 77.54 (9.95) | 75.29 (9.89) | 0.134 |
| Habit of exercise |  |  |  |  | <0.001 |
| No | 57 (70.37%) | 831 (83.69%) | 1207 (86.71%) | 35 (85.37%) |  |
| Yes | 24 (29.63%) | 162 (16.31%) | 185 (13.29%) | 6 (14.63%) |  |
| Drinking status |  |  |  |  | <0.001 |
| Nor or small | 53 (65.43%) | 830 (83.59%) | 1167 (83.84%) | 38 (92.68%) |  |
| Light | 18 (22.22%) | 102 (10.27%) | 164 (11.78%) | 2 (4.88%) |  |
| Moderate | 10 (12.35%) | 61 (6.14%) | 61 (4.38%) | 1 (2.44%) |  |
| Smoking status |  |  |  |  | <0.001 |
| Nor | 32 (39.51%) | 471 (47.43%) | 656 (47.13%) | 26 (63.41%) |  |
| Past | 39 (48.15%) | 273 (27.49%) | 324 (23.28%) | 3 (7.32%) |  |
| Current | 10 (12.35%) | 249 (25.08%) | 412 (29.60%) | 12 (29.27%) |  |

Abbreviations as in Table ​1.
